# MLPA analysis in a cohort of patients with autism

**DOI:** 10.1186/s13039-017-0302-z

**Published:** 2017-02-04

**Authors:** Sara Peixoto, Joana B. Melo, José Ferrão, Luís M. Pires, Nuno Lavoura, Marta Pinto, Guiomar Oliveira, Isabel M. Carreira

**Affiliations:** 1 Cytogenetics and Genomics Laboratory, Faculty of Medicine, University of Coimbra, Coimbra, Portugal; 2 Neurodevelopmental and Autism Unit from Child Developmental Center and Centro de Investigação e Formação Clinica, Hospital Pediátrico, Centro Hospitalar e Universitário de Coimbra, Coimbra, Portugal; 3 Department of Paediatrics of the Centro Hospitalar de Trás-os-Montes e Alto Douro, EPE, Vila Real, Portugal; 4 CNC.IBILI, University of Coimbra, Coimbra, Portugal; 5 CIMAGO - Centro Investigação em Meio Ambiente, Genética e Oncobiologia, Faculty of Medicine, University of Coimbra, Coimbra, Portugal; 6 University Clinic of Pediatrics and Institute for Biomedical Imaging and Life Science, Faculty of Medicine, University of Coimbra, Coimbra, Portugal

**Keywords:** Autism, Autism spectrum disorders, Copy number variants, Genotype, Multiplex Ligation-dependent Probe Amplification (MLPA), Methylation Specific Multiplex Ligation-dependent Probe Amplification (MS-MLPA), Phenotype

## Abstract

**Background:**

Autism is a global neurodevelopmental disorder which generally manifests during the first 2 years and continues throughout life, with a range of symptomatic variations. Epidemiological studies show an important role of genetic factors in autism and several susceptible regions and genes have been identified. The aim of our study was to validate a cost-effective set of commercial Multiplex Ligation dependent Probe Amplification (MLPA) and methylation specific multiplex ligation dependent probe amplification (MS-MLPA) test in autistic children refered by the neurodevelopmental center and autism unit of a Paediatric Hospital.

**Results:**

In this study 150 unrelated children with autism spectrum disorders were analysed for copy number variation in specific regions of chromosomes 15, 16 and 22, using MLPA. All the patients had been previously studied by conventional karyotype and fluorescence in situ hybridization (FISH) analysis for 15(q11.2q13) and, with these techniques, four alterations were identified. The MLPA technique confirmed these four and identified further six alterations by the combined application of the two different panels.

**Conclusions:**

Our data show that MLPA is a cost effective straightforward and rapid method for detection of imbalances in a clinically characterized population with autism. It contributes to strengthen the relationship between genotype and phenotype of children with autism, showing the clinical difference between deletions and duplications.

## Background

Autism is a global neurodevelopment disorder which, in most cases, manifests during the first 2 years and prolongs throughout life, having clinical variances with aging. It belongs to a large family of disorders - autism spectrum disorder (ASD), clinically characterized by difficulties with communication and social interaction, verbal language deficiencies and by repetitive and stereotype behaviour [1]. Epidemiological studies revealed that the number of children with ASD has been increasing throughout the world. In Portugal, a published study in 2007, suggested a prevalence of 9,2/10 000 cases, in the main land, and 15,6/10 000 in the Azores Islands [2]. Several epidemiologic studies identified the importance of genetic factors in autism, specifically the existence of a higher rate of similarities in monozygotic twins (60 to 90%) in contrast to only 3 to 10% in dizygotic twins [3–6]. Structural chromosomic imbalances - copy number variants (CNVs) – seams to be a key player in the disorder and a risk factor for autism especially in the sporadic forms [7–11]. CNVs associated with autism may be inherited or *de novo*, affecting preferentially the chromosomes regions: 1q21, 2p16.3, 3p25-26, 7q36.2, 15q11-13, 15q24, 16p11.2, 16p13.11, 17q12 and 22q11.2 [12–14]. A recent genome wide copy number variation analysis projected between 156 and 280 genomic intervals contributing to autism. Exome sequencing of over 900 individuals provided an estimate of nearly 1.000 contributing genes [15–18]. The most consistently reported submicroscopic chromosome abnormalities detected by chromosomal microarray (10–20%) are recurrent CNVs at 16p11.2, 15q11-13 and 22q11.2 [12, 19, 20]. Submicroscopic rearrangements as deletions or duplications in 15q11-13 region, and especially proximal 15q duplications containing the critical regions of Prader-Willi and Angelman Syndrome (PWS/AS), have been reported in various patients with autism representing the majority of chromosome alterations described in this population [12, 21].

Microdeletions of the short arm of chromosome 16p11.2 were also identified in up to 1% of patients with autism [22–25]. The microduplication of this region was also observed in a similar percentage of individuals, however the association with autism is less convincing due to the increased frequency observed in the control groups [25, 26]. Deletions and duplications in the region 22q13.3 also appear to be risk factors for ASD. Among the three genes (*ACR, RABL2B, SHANK3*) in 22q subtelomeric region, *SHANK3* is the candidate gene for neurobehavioral symptoms observed in affected individuals with 22q13 deletions. This gene has a mutation frequency of 0.5 to 1% in individuals with ASD [27–29].

It is well accepted that array comparative genomic hybridization (CGH) should be the first genetic test to be offered for detection of genomic imbalances in patients with intellectual disability and ASD [30, 31], however access to these costly methods and techniques can be difficult especially given the current economic and financial context in many countries and consequently the pressure from the hospital administrations to contain expense. Clinicians often are confronted with this economical and financial issues and have difficulties to respond to families request to find the cause of these children pathology. One of the aims of this study was to evaluate the detection rate of MLPA technique that could allow a rapid and cost-saving response in a large consultation of children with ASD.

We analysed the presence of CNVs in the most common described chromosomic regions associated with ASD (15q11-q13, 16p11.2 and 22q13) in 150 children using MLPA and MS-MLPA techniques. A clinical assessment of each case was also done to allow genotype-phenotype correlations in the cases with genetic alterations.

## Methods

### Clinical assessment

For this study, a pilot population of 150 children was analysed. They were clinically diagnosed with autism by the Unit of Neurodevelopment and Autism of the Paediatric Hospital, Coimbra Hospital and University Centre, Portugal. The diagnosis was based on a clinical observation by a multidisciplinary team coordinated by a neurodevelopmental paediatrician. ASD diagnosis was assigned on the basis of the gold standard instruments: parental or caregiver interview (Autism Diagnostic Interview-Revised, ADI-R [32]), direct structured proband assessment (Autism Diagnostic Observation Schedule, ADOS [33]), both history and observation for rating (Childhood Autism Rating Scale, CARS [34]) and clinical examination performed by an experienced neurodevelopmental Paediatrician. The latter allows the classification of the degree of autism into mild to moderate (score between 30 and 37) and severe (38–60). The current diagnostic criteria for autism were revised according to the Diagnostic and Statistical Manual of Mental Disorders fifth edition, DSM-5 [1]. It was considered a diagnosis of autism (this term being used synonymously to ASD) any case where ADI-R and ADOS presented as positive scores and all patients met the criteria for ASD from the DSM-5.

An EDTA blood sample from each child was collected for the genetic evaluation by MLPA and MS-MLPA for chromosomes 15q11-13, 16p11 and 22q13. A more detailed clinical evaluation was done for the ten children that presented alterations in the MLPA study, proceeding to the analysis of the relative clinical relevant data to the current clinical history of autism, accordingly to Table 1. Whenever possible, laboratory studies were done on the parents of the children with chromosome alterations.

**Table 1:** Clinical data relevant to the clinical history of the cohort

Personal history: pre and perinatal
Parturition type
Gestational age
Apgar index
Somatometry birth (weight/height/head circumference)
Personal history: Acquisition of neurodevelopment
Walking age
First words
First sentences
Global developmental quotient (GDQ) - Griffiths scale (between 2 and 6 years of age)
Global intelligence quotient (GIQ) - WISC-III (between 6 and 16 years of age)
Pathological personal history
Visual or auditory deficits
Epilepsy (two or more critical episodes in apyrexia)
Family history
Physical exam
Dysmorphisms and signs of neurocutaneous syndromes
Collection of anthropometric measurements (actual growing)
Classical neurologic exam

### Conventional cytogenetic and fluorescence in situ hybridization

All 150 children participating in this study, had been previously evaluated by high resolution conventional cytogenetic using standard GTG-banding on pro-metaphases obtained from 72 h PHA stimulated peripheral blood lymphocyte cultures according to standard procedures [35].

FISH for the critical region 15q11.2 was performed using two commercially available DNA probes: LSI SNRPN/CEP15 (locus D15Z1)/LSI PML and LSI D15S10/CEP15 (locus D15Z1)/LSI PML (Vysis, Chicago, IL) according to standard procedures and manufacturer’s instructions [35].

### Multiplex Ligation-dependent Probe Amplification (MLPA)

**Fig. 1:**
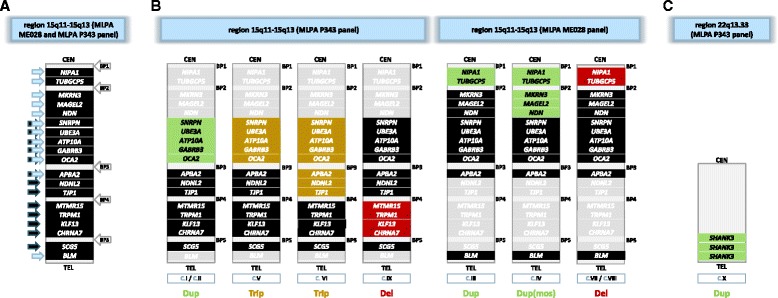
**a** Schematic representation of the studied genes by the two panels (MLPA panel ME028 and panel P343) of probes and of breakpoints on chromosome 15. *Arrows* indicate the genes identified by each panel (P343 – *dark arrows*; MEO28 – *blue arrows*), some of the genes (*two arrows)* are common to both panels; **b** Schematic representation of the extent of alterations detected in the region 15q11-15q13 by the two MLPA panels and the involved genes in the studied cases, with duplications in *green boxes*, triplications represented in *yellow* and deletions in *red*; **c** Schematic representation of the alteration detected in the region 22q13.33 by the MLPA P343 panel. BP-break point; C – Case; CEN-centromere; Del-deletion; Dup-duplication; mos-mosaic; TEL-telomere; Trip-triplication

MLPA (P343-B1) and MS-MLPA (ME028) (Fig. 1a) probe panels were applied as described in the protocols of the manufacturer (MRC – Holland). The P343-B1 panel, applied to all cases, has 54 MLPA probes for the three regions 15q11-13, 16p11 and 22q13, implicated in autism. The 12 most relevant genes studied in chromosome 15 were: *SNRPN-HB2-85, UBE3A, ATP10A, GABRB3, OCA2, APBA2, NDNL2, TJP1, TRPM1, KLF13, CHRNA7, SCG5*). The MS-MLPA ME028 PWS/AS panel, was applied to the patients without alterations detected with the P343-B1 panel. This panel has 25 specific probes for the critical regions of Prader-Willi and Angelman syndromes, as well as five probes to assess the state of methylation, allowing the study of more proximal genes that are not included in P343-B1 panel, like *NIPA1* and *TUBGCP5*. In the region of microdeletion 16p11 the nine studied genes were *LAT, SPN, MAS, MVP, SEZ6L2, HIRIP3, DOC2A, MAPK3, CD2BP2*. The gene *SHANK3* in 22q13 was the one studied in chromosome 22. The products of amplification were identified and quantified by capillary electrophoresis in an ABI 3130 genetic analyser (Applied Biosystems, Japan) and the results were analysed using the GeneMapper v4.1 software (Applied Biosystems, Foster City, USA) and Coffalyser (MRC-Holland, Amsterdam, Holland). The linear ratios of deletion and duplication were fixed at 0.7 e 1.3 respectively.

## Results

The study of the conventional karyotype, together with FISH analysis for 15(q11.2q13) detected duplication of this proximal region in three cases (I, IV, V) and triplication in one case (VI) (Table 2).

**Table 2:** Detailed clinical features of all ten probands with chromosomal abnormalities

Case/Gender	Age (Y)	Diagnosis	Cognition	Family History	Karyotype and FISH (ISCN)	P343 MLPA panel (ISCN)	ME028 MLPA panel (ISCN)	Abnormality origin
I/M	17	ADI-R/ADOS + CARS: 44,5 Severe Autism	Severe/Profound Intellectual Disability (QIG:29)^a^	Brother has autism	46,XY.ish dup(15)(q11.2 q11.2)(SNRPN++,UBE3A++)	rsa15q11.2-q13.1(SNRPN,UBE3A,ATP10A,GABRB3, OCA2)x3	-	Mother normal Father unavailable
II/M	6	ADI-R/ADOS + Severe Autism	Mild Intellectual Disability (QDG:54)^b^	Adopted Biological Mother has Depressive Disorder	46,XY	rsa 15q11.2-q13.1(SNRPN,UBE3A,ATP10A,GABRB3, OCA2)x3	-	unavailable
III/M	6	ADI-R/ADOS + CARS: 35 Mild Autism	No Intellectual Disability (QDG: 102)^b^	Father has drug addiction Brother has social disability	46,XY	rsa(P343)x2	rsa 15q11.2(NIPA1,TUBGCP5)x3mat	Mother
IV/M	12	ADI-R/ADOS + CARS: 30 Mild Autism	Mild Intellectual Disabilty (QDG:58)^b^	Paternal grandfather has schizophrenia Maternal grandmother is alcoholic Paternal Uncle has drug addiction	mos 47,XY,+r.ish r(15)(D15Z4+,SNRPN-,UBE3A-)[69]/r(15)(D15Z4++,SNRPN-,UBE3A-)[2]/46,XY[105]	rsa(P343)x2	rsa 15q11.2(NIPA1,TUBGCP5,MKRN3, MAGEL2,NDN)X3mat	Mother
V/F	9	ADI-R/ADOS + CARS: 37 Moderate Autism	Mild/Moderate Intellectual Disability (QDG:39)^b^	Irrelevant	46,XX,dup(15)(q11.2q11.2)(SNRPN++,D15S10++)	rsa 15q11.2-q13.1(SNRPN,UBE3A,ATP10A,GABRB3, OCA2)x4	-	unavailable
VI/F	10	ADI-R/ADOS + CARS: 32 Mild Autism	Severe/profound Intellectual Disability (QDG:29)^b^	Irrelevant	46,XX,add(15)(q11.2).ish trp(15)(q11.2)(SNRPN,UBE3A)x4	rsa 15q11.2-q13.1(SNRPN,UBE3A,ATP10A,GABRB3, OCA2,APBA2,NDNL2,TJP1)x4dn	-	*de novo*
VII/M	7	ADI-R/ADOS + Mild Autism	No intellectual Disability (QDG: 97)^b^	Irrelevant	46,XY	rsa(P343)x2	rsa 15q11.2(NIPA1, TUBGCP5)x1	unavailable
VIII/M	8	ADI-R/ADOS + CARS: 30 Mild Autism	Intellectual Disability (QDG:71)^b^	Mother has learning difficulties	46,XY	rsa(P343)*x*2	rsa 15q11.2(NIPA1, TUBGCP5)x1mat	Mother
IX/M	14	ADI-R/ADOS + CARS: 30 Mild Autism	Mild Intellectual Disability (QDG:61)^b^	Father has chromosomal abnormalities but no clinical issues Paternal grandmother has Depressive Disorder	46,XY	rsa 15q13.2-q13.3(MTMR15,TRPM, KLF13,CHRNA7)x1pat	-	Father
X/M	5	ADI-R/ADOS + CARS: 31 Mild Autism	No intellectual Disability (QDG:99)	Irrelevant	46,XY	rsa 22q13.33(SHANK3)x3mat	-	Mother

^a^WISC III; M-male, F-female

^b^Griffiths

*ADI-R* Autism Diagnostic Interview Revised

*ADOS* Autism Diagnostic Observation Schedule

*ISCN* International system for human cytogenetic nomenclature, 2016

The application of the MLPA – Panel P343 confirmed two of the cases identified by cytogenetics (I, VI), redefined to a triplication a duplication previously diagnosed by FISH (case V) and identified alterations in three more cases - a duplication (15q11.2-q13.1) (case II) and a deletion (15q13.2-q13.3) (case IX) of the critical region of the chromosome 15 and a duplication in 22q13.33 (case X) (Table 2 and Fig. 1b).

The use of Panel ME028 confirmed one of the alterations, diagnosed by FISH as a mosaic, to be a maternal duplication of the proximal region of 15q11.2 (case IV) and identified three more cases, with normal result after application of MLPA Panel P343: two microdeletions (cases VII and VIII) and one duplication (case III) of the most proximal region of 15q11.2 (*NIPA1, TUBGCP5*) (Table 2 and Fig. 1b).

No alterations were observed in these 150 patients for the critical region 16p11.2.

The main clinical data from these ten patients with structural chromosome alterations are described in Table 3.

**Table 3:** Prenatal and perinatal History and Neurodevelopment

Case	Prenatal History	Actual Growing	GPDD (1st year)	March (months)	1st Words (months)	1st sentences (months)	Epilepsy
I	Part/GA: Forceps/38w BW: 3350 g (P63); BL:52 cm (P90); BHC:35 cm (P72) AI :10	W: P75 H: P75 HC: >P50	No^a^	12	12	Doesn’t build sentences	No
II	Part/GA: Eutocic/ ? BW: 3380 g (P?); AI:10	W: P95 H: P50 HC: P95	Yes	16	12	48	No
III	Part/GA: Ventouse/39w BW: 3450 g (P55); BL:50 cm (P47); BHC:35.5 cm (P69); AI :10	W: P90 H: P75 HC: >P50	No^a^	12	24	36	No
IV	Part/GA: CS/ 40w BW: 3460 g (P41); BL:50.5 cm (P39); BHC:35 cm (P45); AI:10	W: P75/90 H: P90 HC: P90	Yes	18	14	36	No
V	Part/GA: Ventouse/39w BW: 2485 g (P4); BL:47 cm (P8); BHC:32.5 cm (P11) AI :10	W: P25 H: P25 HC: P50	Yes	24	20	36	No
VI	Part/GA: Eutocic/? BW: 2840 g (P?); BL:50 cm (P?); IA:10	W: >P75 H: P50 HC: P50	Yes	24	60	Doesn’t build sentences	Yes
VII	Part/GA: Eutocic/35w BW: 2510 g(P46); BL:45 cm (P31); BHC:33 cm (P72) AI:10	W: P50 H: P50 HC: P75	Yes	24	36	42	No
VIII	Part/GA: CS/ 38w High Risk in Pregnancy due to previous abortions (2) BW: 3470 g (P72); BL:49 cm (P47); BHC:36 cm (P88); AI:10	W: P95 H: P95 HC: > > P50	Yes	15	40	60	No
IX	Unknown	W: P50 H: P50 HC: >P95	Yes	19	30	42	No
X	Part: CS/ 41w BW: 4610 g (P94); BL:51 cm (P35); BHC:37 cm (P76); AI:10	W: P95 H: P90 HC: >P95	No^a^	12	12	36	No

^a^only identified at the age of 2 years old - followed by regression

Pre and perinatal history: *AI* Apgar Index at 5 min, *BHC* Birth head circumference, *BL* Birth length, *BW* Birth Weight, *CS* Caesarean Section, *GA* Gestational Age, *Part* Parturition type, *P* percentile according Fenton growth chart

Actual Growing: *HC* head circumference, *H* Height, *W* – Weight

*GPDD* Global psychomotor developmental delay, *w* pregnancy weeks, *g* grams, *cm* centimeters

The study of the patient’s parents was possible in only six of the ten individuals with alterations: in four of them, the alteration is maternally inherited (cases III, IV, VIII and X), one has a paternal origin (case IX) and in case VI the alteration is *de novo*. For the remaining four cases, it was not possible to ascertain the parental origin because either child was adopted or because some parents rejected to be studied.

Cases I and II, are carriers of the same duplication on chromosome 15 [15q11.2q13.1(SNRPN,UBE3A,ATP10A,GABRB3,OCA2) x3] (Fig. 1b), both have severe autism but with different levels of cognition. Case II is an adopted boy with a global psychomotor developmental delay from the first year of age and currently with mild intellectual disability.

Cases III and IV, evaluated by the second MLPA probe panel have more proximal duplications of chromosome 15 (Fig. 1b). Both children have mild autism. Case III which has the more proximal duplication shows a normal global intelligence quotient (GIQ) and a normal motor and language development. Case IV has a mild intellectual deficit.

Cases V and VI (Fig. 1b), both presented mild to moderate autism but with distinct levels of intellectual disability, varying from mild in case V to severe in case VI, which is coincident with the extent of the triplicated region that includes a greater number of genes. In both patients, strabismus is present as well as motor development delay, not found in previously described patients, carriers of duplications. This strongly suggests a gene dosage effect.

Cases VII and VIII (Fig. 1b) both have microdeletions of the proximal genes, both have similar levels of neurodevelopment features, presenting mild autism and mild (case VIII) or absent (case VII) intellectual disability. In case VIII, the alteration is of maternal origin and the progenitor is reported as having learning difficulties.

In case IX (Fig. 1b), the deleted region is distal to the previous two cases reported and involves a greater number of genes, however the clinical presentation is similar between the three cases (VII, VIII and IX). In this case the deletion was inherited from the father, but was not possible to ascertain his phenotype.

Lastly, from the 150 individual evaluated there was one case (X) identified with duplication of the SHANK3 gene on 22q13.33, associated with mild autism and average intellectual quotient and without other significant neurodevelopmental alterations except for clumsiness. The duplication was inherited from an asymptomatic mother.

Concerning to the prenatal history, most cases did not reveal any anomalies, being born from gestations of term, without prenatal incidents. As for the early development markers, half of the children walked after 18 months, which suggests an early alteration in neurodevelopment. However, the growth with regards to stature, weight and head circumference, did not reveal alterations (Table 3).

## Discussion

**Fig. 2:**
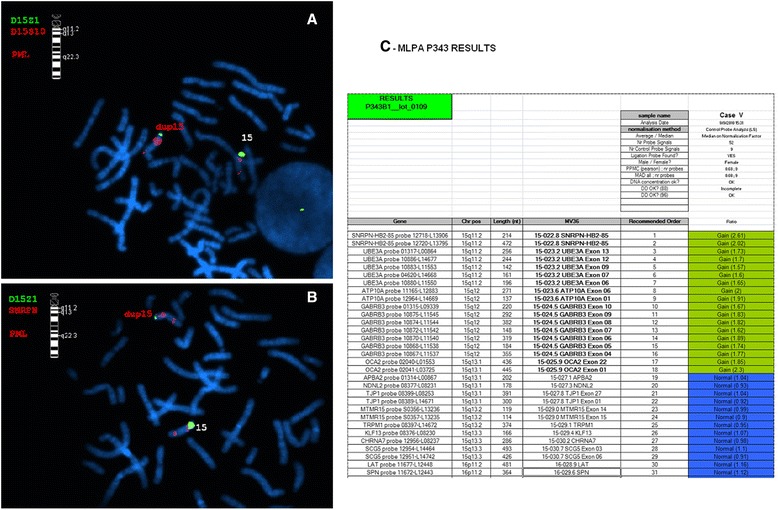
FISH and MLPA results for patient V. **a** Metaphase hybridization showing a gain in 15q11.2 (D15S10 probe in *red*) interpreted as a duplication. **b** Metaphase hybridization showing a gain in 15q11.2 (SNRPN probe in *red*) interpreted as a duplication. **c** Overview of MLPA P343 result using CoffalyserV7 software (MRC Holland, Amsterdam, Netherland) revealing the presence of 4 copies and not 3 of region 15q11.2q12 in patient V compatible with a triplication and not a duplication

Of the 150 children evaluated in this pilot study, only four (3%) had revealed alterations detected by conventional cytogenetics and FISH. This number increased after MLPA and MS-MLPA analysis with the identification of 6 more imbalances. Being a technique with higher resolution, MLPA also allowed the redefinition of a duplication, initially reported by conventional cytogenetics as a triplication (case V - Fig. 2) and allowed the identification of imbalances of six new cases (Table 2).

Of these ten patients, only one presented duplication on the *SHANK3* gene on 22q13.33, which is in accordance with the reported frequency in the population (1% of patients with ASD) [13, 28]. All the others showed imbalances on chromosome 15 (four duplications, two triplications and three deletions) [9, 36, 37]. No cases of deletion nor duplication on 16p11.2 were found in our sample, possibly due to the relative low frequency of up to 1% mentioned in other studies. Walsh and Bracken in 2011 made a review of literature and a meta-analysis of 3613 ASD patients from seven studies redefining a prevalence of 16p11.2 microdeletion CNVs in 0.5% (0.31–0.82%, 95% CI) and 16p11.2 microdeletion CNVs in 0.28% (0.14–0.56%, 95% CI) not in disagreement with our results [25, 38].

### CNVs in 15q11-15q13 region

In cases I, II, III and IV we found duplications of specific regions of chromosome 15 that involve several break points (Fig. 1b).

Two of the patients (case I and II) presented a duplication that affects BP2-BP3 (Fig. 1b). This alteration involves the critical region for Prader-Willi and Angelman Syndrome which is subjected to genomic imprinting and referred as the imbalance more frequently found in individuals with ASD [37, 39]. Numerous studies support that the majority of cases with this alteration are associated to maternal transmission or appear *de novo*, while the paternal inheritance leads to a normal phenotype [37, 40, 41]. It was not possible to ascertain the parental origin in these two patients (cases I and II), however the family history is suggestive of neurodevelopmental delays, with a brother with autism (case I) and neuropsychiatric disorders, with mother with depressive disorder (case II), indicating a possible inheritance of the mentioned alterations (Table 2). These two cases (I and II) with the same genetic imbalances, both have severe autism although with different intellectual levels. According to Bolton PF et al. (2001) and Depienne C et al. (2009) the patients with duplication of the critical region (BP2-BP3), as occurs in these two cases, have frequently an abnormal phenotype that includes development delay, especially in speech and language, different degrees of intellectual disability, motor coordination difficulties and mild dysmorphisms (which are absent in our cases) [34, 40].

Case III, is a case of a more proximal duplication (between BP1-BP2) (Fig. 1b). The patient has mild autism, without intellectual impairment and with normal motor and language development. The common phenotype found in this alteration is highly variable, even between members of the same family [40, 42]. It can vary between autism, other neurodevelopmental disorders, mild socialization problems or learning difficulties, neuropsychiatric manifestations or even a normal phenotype, which is corroborated by other authors [37, 41]. The genetic alteration in this patient is also present in his mother who is phenotypically normal. One of the patient’s brother, presents a serious socialization problem nevertheless he was not available for study.

Case IV also presents a duplication of the proximal region involving more genes than the previous one (case III) (Fig. 1b). It corresponds to a mosaicism with three cell lines. This patient presents mild intellectual disability and mild autism, as in case III. In this case IV, a milder phenotype could be expected since this aberration is present in a mosaic state. In the blood lymphocytes, the normal cellular lineage is the most representative. The ring chromosome was inherited from the mother that is not a mosaic in the blood and does not present with any type of clinical alteration. Other tissues were not evaluated for the detection of the mosaic state.

Regarding the two cases with triplication (V and VI), both have mild to moderate autism with distinct levels of intellectual disabilities which is, mild in case V but severe in case VI. The latter have a greater number of triplicated genes, being the distal breakpoint correspondent to BP4 (Fig. 1b). Genes *APBA2, NDNL2* and *TJP1* that are triplicated in case VI may be responsible for the more severe intellectual disability. Both patients had delay motor developmental skills (age of walking 24 months), which was not seen in the cases with duplication in our cohort, suggesting that a greater number of copies may be related to a more severe motor development delay. Both present vision deficit and one of them (case VI) also has epilepsy, which has been frequently associated with autism [43, 44].

Cases VII and VIII present microdeletion of proximal genes (*NIPA1, TUBGCP5*) and show similar levels of motor and cognitive development, both have mild autism with normal intelligence quotient and motor and language development delay, noted from the first year. These discrete phenotypes, as it happens in cases III and IV, that involve duplications of the same region, suggests that alterations involving this region, that not include the critical region of PWS and AS may not have any significant clinical effects. These alterations may also manifest as a milder form, like the mother in case VIII that, despite having the exact same deletion as her son, only had learning difficulties in her childhood which did not affect her adult independent life. Cases like this had been described repeatedly with significant phenotypic variability [39, 45]. Copy numbers losses and gains in this region have been identified with a frequency of 1% in the normal population, proving to be a challenge for genetic counselling [37, 39].

Case IX also corresponds to a microdeletion that is different from the previous ones since there are other breakpoints involved (BP4-BP5) (Fig. 1b) with a greater number of genes involved (*MTMR15, TRPM1, KLF13, CHRNA7*). The clinical condition is however similar to the two previous cases, contrary to what would be expected, based on the genetic differences. In this case, the deletion was inherited from an apparently normal father. This situation has already been reported in other cases, supporting the evidence of a higher frequency of deletions inherited from healthy parents, comparing to those that occur *de novo* [46, 47]. There are a great variety and heterogeneity of the phenotypic expression for this deletion of 1.6 Mb size. It ranges from normal phenotype (as it happens in the father), to intellectual disability with borderline intellectual quotient and autism (present in the patient), epilepsy, bipolar disorder, schizophrenia and other neuropsychiatric disorders [47, 48]. Depression that are frequently associated with this deletion, has been refered in the patient paternal grandmother but she was unavailable for study. In our sample, no cases of duplication of BP4-BP5 were found, which is in agreement with other reports [48–50]. If duplications involving this region cause phenotypical anomalies, its penetrance seems to be lower than it is in deletions which therefore may explain its minor frequency.

The structural alterations presented in this study involving various regions of chromosome 15, have a significant role in the pathogenesis of other different conditions. They affect predominantly the brain function like in ASD and intellectual disability (that varies from mild to severe), epilepsy and neuropsychiatric disorders like schizophrenia and bipolar disease. This phenotypic variability suggests that other events may contribute for the manifestation of those conditions.

Various factors have been proposed to explain this variability in phenotype. Besides from incomplete penetrance and the contribution of adjacent genes, the presence of a masked recessive mutation or functional polymorphism on one of the genes may be another factor, environmental and perinatal factors, maternal conditions [51, 52]. The possibility of different phenotypic expressions between diverse members of the family with a deletion, caused by defects of imprinting, would be another hypothesis to explain this variability, however it seems unlikely on the alterations that affect BP1-BP2 and BP4-BP5, as none of the genes in these regions have been described as imprinted (Genomic Imprinting Website: www.geneimprint.com/site/genes-by-species, September 2008).

Another hypothesis is the possible capability that some individuals may have to overcome one or more deficiencies caused by haploinsufficiency of specific genes during pre or postnatal development [53]. This supports the fact that some carriers have normal development from birth, while others have learning difficulties during their childhood that cause no impact in the adult life. This is enforced by the fact that some parents who present the same alterations as their children, have no problem related to social integration. In addition to all these possible explanations, one cannot exclude the presence of other CNVs not detected with this approach, neither the environmental factors that may also play a vital role in the child’s development.

### CNVs in SHANK3 at 22q13.33 region

A single duplication was found on gene *SHANK3* at 22q13.33 which is associated to mild autism with normal intellectual quotient. Microdeletions and point mutation involving *SHANK3* region have been reported as cause of a spectrum of neuropsychiatric disorders including “22q13 deletion syndrome” (also known as Phelan-Mcdermid syndrome) and ASD [28, 29]. In the same way, 22q13 duplications involving *SHANK3* were reported in patients with Asperger syndrome, attention deficit-hyperactivity disorder (ADHD) or schizophrenia, suggesting that an overexpression of this gene could be also pathogenic [27]. In our sample, case X is a patient with a *SHANK3* duplication that was inherited from a healthy mother which leads us to question of its pathogenicity. A situation of incomplete penetrance or expression variability cannot be ruled out in the same way as in 22q11.2 duplication syndrome [54], neither can be excluded the presence of other CNVs not detected with this approach.

## Conclusion

In this study, it was also possible to establish some correlations between certain genotypes and corresponding phenotypes, despite of some variability.

The study of autism is a challenge due to the great variety of behavioural and neurodevelopmental manifestations with variable severity and many co-morbidities. With the development of new technologies that allow an approach to the whole genome, certainly there will be a greater contribution for a better comprehension of its aetiology. It is vital that children with ASD are followed-up by specialized professionals in the neurodevelopment area, so that clinical details can be well accurate to allow strong genotype-phenotype correlations.

Our study indicates that MLPA can be a cost-effective method for detection of microdeletions and microduplications in ASD population. In patients with relevant phenotypic characteristics, this approach could replace other expensive and laborious techniques in clinical diagnosis. Additionally, in some cases karyotype and/or FISH analysis should be considered, mainly for the detection of supernumerary marker chromosomes. For example, the invdup(15) is a well documented cause of ASD with distinct phenotype and prognosis.

It is well established that array-CGH should be the first-tier test for neurodevelopmental disorders, MLPA, although with some limitations, since it is a target method, can be considered as a test in large populations where the costs and economic pressures limits a more expensive method for diagnosis.

## Abbreviations

ADHD: Attention deficit-hyperactivity disorder
ADIR: Autism Diagnostic Interview – Revised
ADOS: Autism Diagnostic Observation Schedule
AS: Angelman Syndrome
ASD: Autism spectrum disorder
BP: Break point
CARS: Childhood Autism Rating Scale
CGH: Comparative genomic hybridization
CNV: Copy number variants
DSM-5: Diagnostic and Statistical Manual of Mental Disorders
EDTA: Ethylenediaminetetraacetic acid
FISH: Fluorescence *in situ* hybridization
GIQ: Global intelligence quotient
MLPA: Multiplex ligation dependent probe amplification
MS-MLPA: Methylation specific multiplex ligation dependent probe amplification
PWS: Prader Willi Syndrome
